# Novel developments in the pathogenesis and diagnosis of extranodal marginal zone lymphoma

**DOI:** 10.1007/s12308-017-0302-2

**Published:** 2017-09-25

**Authors:** Max I. Schreuder, Michiel van den Brand, Konnie M. Hebeda, Patricia J. T. A. Groenen, J. Han van Krieken, Blanca Scheijen

**Affiliations:** 1 Department of Pathology, Radboud University Medical Center, Geert Grooteplein Zuid 10, 6525 AG Nijmegen, The Netherlands; 2 Pathology-DNA, Rijnstate Hospital, Arnhem, The Netherlands; 3 Radboud Institute for Molecular Life Sciences, Nijmegen, The Netherlands

**Keywords:** Lymphoid malignancies, Chromosomal rearrangements, Infections, MALT, EMZL

## Abstract

Extranodal marginal zone lymphoma (EMZL), mostly represented by mucosa-associated lymphoid tissue (MALT) type, also referred to as MALT lymphoma, is a clinically heterogeneous entity within the group of low-grade B cell lymphomas that arises in a wide range of different extranodal sites, including the stomach, lung, ocular adnexa, and skin. It represents the third most common non-Hodgkin lymphoma in the Western world, and the median age of occurrence is around 60 years. One characteristic aspect in a subset of EMZL detectable in about 25% of the cases is the presence of specific chromosomal translocations involving the genes *MALT1* and *BCL10*, which lead to activation of the NF-κB signaling pathway. Another unique aspect is that several infectious agents, such as *Helicobacter pylori* in the case of gastric EMZL, and autoimmune disorders, like Sjögren syndrome, have been implicated in the pathogenesis of this cancer. Recent findings as summarized in this review have further improved our understanding of the complex pathobiology of this disease and have been essential to better define novel treatment strategies. In addition, many of these specific features are currently being implemented for the diagnosis of EMZL.

## Introduction

There are three different types of marginal zone lymphomas (MZLs): (i) extranodal marginal zone lymphoma (EMZL), mostly represented of mucosa-associated lymphoid tissue (MALT) type; (ii) splenic MZL (SMZL); and (iii) nodal MZL (NMZL). EMZL accounts for about 7% of all adult non-Hodgkin lymphoma (NHL) and 70% of MZL [1]. The most predominant site for EMZL involves the stomach (70%), but virtually all other organs can be affected, including the lung, salivary gland, ocular adnexa, skin, and thyroid. Despite their clinical heterogeneous presentation, at least three common variants of chromosomal translocations have been identified as specific for EMZL, all of which affect the NF-κB pathway [2]. Moreover, EMZLs are frequently associated with chronic inflammation and infectious agents that give rise to chronic infections, such as *Helicobacter pylori* in gastric EMZL, *Chlamydophila psittaci* in ocular adnexa EMZL, *Campylobacter jejuni* in immunoproliferative small intestinal disease (IPSID), and *Borrelia burgdorferi* in cutaneous EMZL [3]. On the other hand, several autoimmune disorders, including Sjögren syndrome, lymphoepithelial sialadenitis and Hashimoto thyroiditis, predispose to EMZL development. The prevailing view is that continuous immune stimulation resulting from chronic infections and autoinflammatory diseases cooperates with recurrent genetic aberrations resulting in lymphoid transformation.

EMZL, in general, shows a remarkably indolent disease course with a median survival of more than 12 years [4]. However, in a small proportion of cases, EMZL can progress and undergo histological transformation into aggressive high-grade tumors, mostly diffuse large B cell lymphoma (DLBCL) [5]. A common feature of EMZL is deregulation of the proteolytic activity of the MALT1 protein, which results in constitutive nuclear factor κB (NF-κB) stimulation. Current and novel therapeutic strategies are aimed to target these specific features underlying the molecular pathogenesis of EMZL. In this review, novel insight into molecular pathogenesis of EMZL will be described and its impact on diagnosis and therapy of this disease spectrum.

## Clinical features of EMZL

EMZL often occurs in organs devoid of prominent organized lymphoid tissue, where as a result of chronic inflammation, outgrowth of a malignant clone progressively replaces the reactive lymphocyte population. Irrespective of the site of origin, EMZL is characterized by an indolent presentation and course, mainly occurring in adults with a median age of 60 years. The clinical presentation differs depending on the organ involved. Patients with gastric EMZL often present with symptoms that mimic those of peptic ulcer disease or gastritis (nausea, dyspepsia, and chronic fatigue), while recurrent respiratory infections, chest pain, and dyspnea are observed in patients with pulmonary EMZL. Patients with conjunctival EMZL may present with blurry vision or other visual field defects. The majority of the patients with EMZL display localized stage I or II extranodal disease (Ann Arbor staging system), involving epithelial tissues at specific sites, including the gastrointestinal tract. In about 30% of the cases, these lymphomas disseminate to other MALT sites, predominantly lymph nodes and in very rare cases to the bone marrow, but the peripheral blood is usually not involved [6]. The outcome of patients with EMZL is good with a 5-year overall survival between 86 and 95%, without any significant differences between the site of the EMZL, localized or disseminated disease [7].

## Pathogenesis of EMZL

**Fig. 1:**
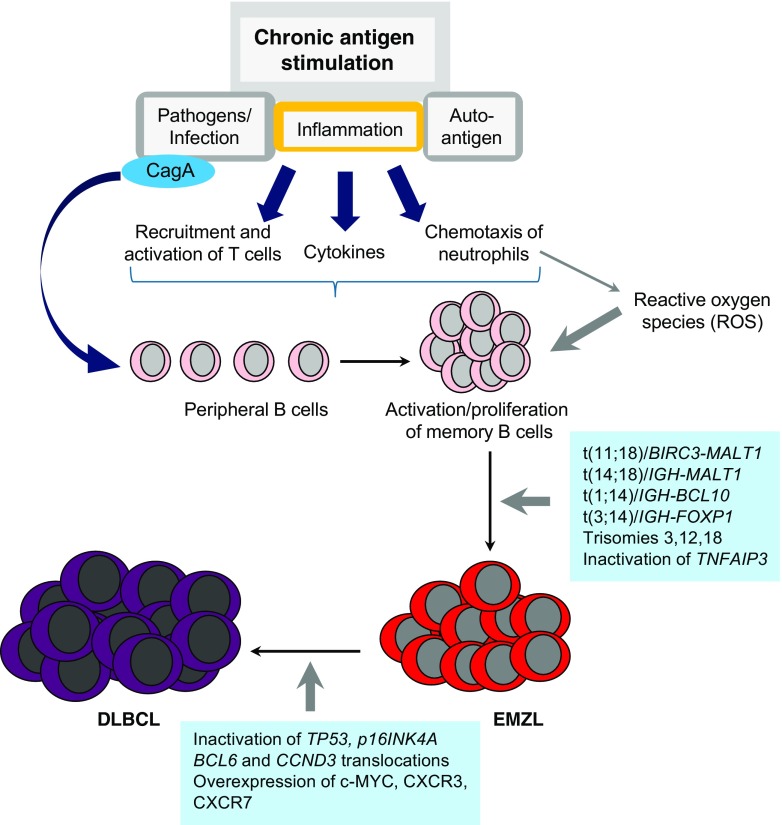
Pathogenesis of extranodal marginal zone lymphoma. At the site of chronic antigen stimulation there is inflammation resulting from infection with specific pathogens (e.g., *Helicobacter pylori*) or the response to autoantigens present in autoimmune disorders, like Sjögren syndrome and Hashimoto thyroiditis. This results in recruitment and activation of T cells, production of proinflammatory cytokines, chemotaxis of neutrophils releasing reactive oxygen species (ROS), and production of the cytotoxin-associated gene A (CagA) protein harboring oncogenic properties in case of *Helicobacter pylori* infection. The continuing antigenic stimulation causes a polyclonal activation and expansion of B cells in the context of specific antigens. Due to the increased proliferation rate, stimulation of different receptor signaling pathways, like B cell receptor (BCR), Toll-like receptors (TLR), B cell-activating factor (BAFF) collectively activating NF-κB, and exposure to DNA damaging effects of ROS, genomic aberrations can occur that promote the development of extranodal marginal zone lymphoma (EMZL). These include t(11;18), t(14;18), t(1;14), and t(3;14) translocations; trisomy of chromosomes 3, 12, and 18; and inactivation of *TNFAIP3*. The indolent growth characteristics of EMZL may be altered, due to transformation to the more aggressive diffuse large B cell lymphoma (DLBCL). This is facilitated by inactivation of the tumor suppressor genes *TP53* and *p16INK4A*, translocations involving oncogenes *BCL6* and *CCND3*, and upregulation of c-MYC, CXCR3, and CXCR7

The term “marginal zone lymphoma” refers to the fact that these lymphoma cells are derived from post-germinal center memory B cells normally present in the marginal zone of lymphoid organs. In nearly all cases, EMZL displays fully rearranged immunoglobulin heavy chain variable (IGHV) and light chain genes, which show somatic hypermutation and class switching [8, 9]. In many cases, EMZL has been shown to be associated with chronic immune reactions driven by bacterial, viral, or autoimmune stimuli (Table 1). This latter aspect correlates with the observation that patients with autoimmune disorders harbor an increased risk for the development of lymphomas [10, 11]. These findings have led to the hypothesis that this type of indolent lymphoma follows a multistage development that starts with an infection combined with (auto-)antigenic stimulation or other direct effects on B cells, like the presence of free radicals in an inflammatory surrounding. With the subsequent accumulation of genetic alterations, which frequently result in activation of the NF-κB pathway, neoplastic transformation can occur, decreasing the dependency of antigenic stimulation (Fig. 1). Nonetheless, many of the EMZL show regression upon eradication of the bacterial infections with specific antibiotic treatment, which is mainly the case in translocation-negative EMZL.

**Table 1:** Summary on the main characteristics of extranodal marginal zone lymphoma (EMZL)

Primary site	% EMZL	Infection/autoimmunity	Genetic alterations
Stomach	70	*Helicobacter pylori* (85%) *Helicobacter heilmannii* (< 1%)	t(11;18)(q21;q21)/*BIRC3-MALT1* (23%) t(3;14)(p14;q32)/IGH*-FOXP1* (3%) t(1;14)(p22;q32)/IGH*-BCL10* (2%) t(14;18)(q32;q21)/IGH*-MALT1* (1%) *TNFAIP3* inactivation (5%)
Salivary gland	9	Lymphoepithelial sialadenitis/ Sjögren syndrome (20–45%) Hepatitis C virus (30%)	t(14;18)(q32;q21)/IGH*-MALT1* (6%)) t(11;18)(q21;q21)/*BIRC3-MALT1* (2%) t(1;14)(p22;q32)/IGH*-BCL10* (1%) *TNFAIP3* inactivation (8%)
Ocular adnexa	7	*Chlamydophila psittaci* (10–50%)	t(3;14)(p14;q32)/IGH*-FOXP1* (20%) t(14;18)(q32;q21)/IGH*-MALT1* (16%) t(11;18)(q21;q21)/*BIRC3-MALT1* (7%) *TNFAIP3* inactivation (38%)
Lung	4	*Achromobacter xylosoxidans* (40%)	t(11;18)(q21;q21)/*BIRC3-MALT1* (45%) t(1;14)(p22;q32)/IGH*-BCL10* (8%) t(14;18)(q32;q21)/IGH*-MALT1* (7%) *TNFAIP3* inactivation (9%)
Skin	4	*Borrelia burgdorferi* (20%)	t(3;14)(p14;q32)/IGH*-FOXP1* (10%) t(14;18)(q32;q21)/IGH*-MALT1* (7%) t(11;18)(q21;q21)/*BIRC3-MALT1* (4%)
Intestinal tract	2	*Campylobacter jejuni* (50%)	t(11;18)(q21;q21)/*BIRC3-MALT1* (19%) t(1;14)(p22;q32)/IGH*-BCL10* (7%)
Thyroid	2	Hashimoto thyroiditis (90%)	t(3;14)(p14;q32)/IGH*-FOXP1* (50%) t(11;18)(q21;q21)/*BIRC3-MALT1* (9%) *TNFAIP3* inactivation (11%)

### Bacterial infections

#### *Helicobacter pylori*

*H. pylori* infection is present in 85–90% of gastric EMZL, and support for its role as an etiologic factor was provided in the early 1990s after demonstration of tumor regression in the early-stage cases treated with antibiotic therapy. Although *H. pylori* infection can be detected in about 50% of the general population giving rise to chronic active gastritis or even peptic ulcer disease, only ~ 1% of the infected subjects will develop gastric adenocarcinoma or lymphoma. A population-based study has demonstrated a declined incidence of gastric EMZL after specific intervention for *H. pylori* infections in patients with acid peptic disease symptoms [12].

More direct support for the role of *H. pylori* in the pathogenesis of gastric EMZL derives from studies that have shown that gastric EMZL cell growth could be stimulated in culture by *H. pylori*-specific T cells [13]. An additional effect of *H. pylori* on the microenvironment is the release of the proliferation-inducing ligand (APRIL) by lymphoma-associated macrophages [14]. Furthermore, the *H. pylori* cytotoxin-associated gene A (CagA) protein has direct oncogenic properties both for gastric epithelial cells and B lymphocytes [15, 16]. The CagA protein can enter B cells via type IV secretion system in an ATP-dependent manner [17], where it undergoes tyrosine phosphorylation by SRC or ABL kinases in the C-terminal region [18, 19]. Phosphorylated CagA interacts with Grb2 and tyrosine phosphatase SHP-2 leading to ERK activation [20], which promotes phosphorylation of the pro-apoptotic protein BAD and upregulation of the anti-apoptotic molecules BCL2 and BCL-X_L_[17, 21]. Detection of CagA, phospho-SHP2, and phospho-ERK predicts involvement and dependence of *H. pylori* in the pathogenesis of gastric EMZL [22]. Alternatively, CagA can block cell cycle progression and inhibits B lymphocyte apoptosis by impairing the JAK/STAT and p53 pathway [23, 24]. Furthermore, *H. pylori* activates the NF-κB pathway in lymphocytes through both the canonical and non-canonical pathways [25]. These findings provide further evidence that gastric EMZL follows a multistage progression from chronic gastritis to gastric lymphoma that starts with *H. pylori* infection.

#### *Helicobacter heilmannii*

Additional non-*H. pylori* species have been identified in human gastric mucosa, now reclassified as *Helicobacter heilmannii* sensu lato (*H. heilmannii* s.l.) without specific sequence information; and *Helicobacter heilmannii* sensu stricto (*H. heilmannii* s.s.) or any of the other ten species names if definite identification at the species level is achieved [26]. The frequency of human *H. heilmannii* s.l. infection is less than 1% of the population in industrialized countries and 3–8% in developing countries. Similar to *H. pylori, H. heilmannii* s.l. infection has been associated with gastritis, peptic ulcer disease, gastric carcinoma, and gastric EMZL [27]. However, it seems that there is a relatively higher prevalence of gastric EMZL in patients with *H. heilmannii* s.l. gastritis, i.e., 2% in comparison to 0.7% among patients with *H. pylori* gastritis [28].

#### *Chlamydophila psittaci*

The *Chlamydophila* genus is the etiologic agent of psittacosis, also known as parrot disease, an infection caused by exposure to infected bird species. *C. psittaci* was recognized as a potential trigger of ocular adnexal lymphoma, when Ferreri et al. showed the efficacy of antibiotic treatment [29, 30]. *C. psittaci* DNA has been detected in a variable percentage of ocular adnexal lymphoma, with a high incidence of 47 to 80% in especially Italy, Austria, Germany, and Korea, but with a much lower incidence in UK and Southern China [31], while there was no evidence of *C. psittaci* infection in cases from the USA and Japan [32–34]. However, support for its role as a causative agent in ocular adnexal lymphoma has been provided by the findings of detecting chlamydial antigens in tumor biopsies and the isolation of chlamydia from conjunctival swabs and peripheral blood from lymphoma patients as well as the visualization of *C. psittaci* within tumor-infiltrating macrophages by electronic microscopy [35].

#### *Campylobacter jejuni*

The Gram-negative helical-shaped *Campylobacter jejuni*, which is usually carried by birds, represents one of the most common causes of gastroenteritis in the world. Persistent infection leads to severe gastrointestinal illness, which requires antimicrobial therapy, including macrolides and fluoroquinolones. *C. jejuni* is also an initiating factor in chronic autoimmune disease, such as Guillain-Barrè syndrome and reactive arthritis [36]. *C. jejuni* has also been associated with the pathogenesis of immunoproliferative small intestinal disease (IPSID), a special subtype of EMZL that primarily occurs in young adults of the Middle East, North and South Africa, and the Far East. The presence of *C. jejuni* DNA has been demonstrated in a small cohort of IPSID samples [37], and clinical response to antibiotics directed at this infection has been described in a single study [38].

#### *Borrelia burgdorferi*

The spirochete *Borrelia burgdorferi* is a tick-borne obligate parasite and infection of humans can result in Lyme borreliosis. Moreover, *Borrelia* infection has been linked to cutaneous EMZL with higher detection rates in endemic areas, such as the Scottish Highlands and Austria [39, 40]. In Europe, the association varies between 10 and 42% and is almost absent in non-endemic areas [41]. However, even in non-endemic regions, like France, *B. burdorferi* DNA is detected in 19% of the cases with primary cutaneous EMZL [42].

#### *Achromobacter xylosoxidans*

Primary lymphoma of the lung is a rare entity representing ~ 4% of all extranodal lymphomas and 0.4% of NHL. Although pulmonary parenchyma is devoid of organized lymphoid tissue under normal physiological conditions in adults, it develops due to some disease entities, like pulmonary inflammatory process, follicular bronchiolitis, and acute infections. In one report, *Achromobacter xylosoxidans*, a Gram-negative bacterium with low virulence but high resistance to antibiotic therapy, has been detected with a significantly increased prevalence in patients with pulmonary EMZL as compared to non-lymphoma biopsies [43].

### Viral infections

#### Hepatitis C virus

Belonging to the *Flaviviridae* family of RNA viruses, hepatitis C virus (HCV) infects both hepatocytes and lymphocytes and is strongly linked to the pathogenesis of hepatocellular carcinoma and B cell NHL, including MZL. Analysis on risk factors in EMZL has clearly established an increased risk associated with HCV seropositivity, and HCV infection has been documented in about one-third of patients with non-gastric EMZL [44]. The causal relationship between HCV and EMZL is further substantiated by the observation of lymphoma regression after antiviral treatment [45]. EMZL in HCV-infected patients most often occurs at non-gastric sites, especially the salivary and lacrimal glands. The proposed underlying mechanisms for HCV-associated EMZL include a direct oncogenic effect of HCV-encoded proteins, an indirect antigen-driven stimulation, or immune suppression [46].

### Autoimmune disorders

#### Sjögren syndrome

Primary Sjögren syndrome (pSS) is a complex autoimmune disease that includes lacrimal and salivary gland disease, serum antibodies like anti-SSA, anti-SSB, rheumatoid factor, and salivary duct antibodies [47]. Consequently, in more than 20–40% of the patients the disease extends beyond the exocrine glands, manifested either by epithelial lymphocytic infiltration of the lungs, liver, or kidney or by immune complex-mediated phenomena such as skin vasculitis, peripheral neuropathy, and glomerular nephritis [48]. The incidence rate of pSS is 7 cases per 100,000 person-years and occurs most frequently in the fourth to seventh decades of life affecting more women than men. In patients with pSS, there is a 15-fold increased incidence of NHL that affects 5–10% of these patients, especially EMZL of the salivary glands [49, 50]. Notably, translocations involving *MALT1* occur less frequently in EMZL of pSS patients [51]. However, germline mutations in BAFFR (*TNFRSF13C*) as well as germline and somatic coding variant of *TNFAIP3* (*A20*) have been linked to increased risk of pSS and associated lymphoma [52, 53].

#### Lymphoepithelial sialadenitis

Lymphoepithelial sialadenitis (LESA) is a benign lymphocytic infiltration of salivary gland tissue producing atrophy of the columnar ductal epithelium. In addition, there is intraepithelial infiltration of monocytoid B cells or centrocyte-like cells, which promotes proliferation of basal epithelial cells and lymphoepithelial lesions [54]. LESA is an autoimmune lesion and a component of Sjögren syndrome, but can also occur without Sjögren syndrome. The lymphoid infiltrate has a predominance of T cells, but within the foci of epithelial proliferation, lymphocytes have features of marginal zone B cells. In some cases, these foci display clonal IG rearrangements, but without evidence of progressive expansion [55]. LESA lesions are frequently controllable with corticosteroid treatment, but can progress to salivary EMZL.

#### Hashimoto thyroiditis

Hashimoto thyroiditis (HT) is a common form of autoimmune thyroid disease affecting up to 2% of the general population, and more prevalent in women than men. Longstanding autoimmune HT has been directly linked to primary thyroid EMZL, which is quite a rare neoplasm accounting for 2–8% of all thyroid malignancies and 2% of all extranodal lymphomas [56]. Among patients with HT, there is a 60-fold increased risk of thyroid EMZL that affects 0.5% of the patients. The key factor in the development of HT is breakdown of immune tolerance, initiated by inflammatory events in the gland probably as a result of viral or bacterial infection or injury to the thyroid cells from toxins like iodine [57]. The injured thyroid cells may exhibit new epitopes, resulting in an influx of antigen presenting cells, clonal expansion of autoreactive T cells, and IgG producing B cells. The development of lymphoid tissue directly in the thyroid gland with progressive destruction of the thyroid cells, eventually leads to hypothyroidism [58]. The molecular pathways that contribute to lymphoma progression in HT remain to be identified, but it is interest to note that translocations involving *FOXP1* occur at a relative high frequency in thyroid EMZL [59].

## Genetic alterations present in EMZL

### Chromosomal aberrations and gene deletions

**Fig. 2:**
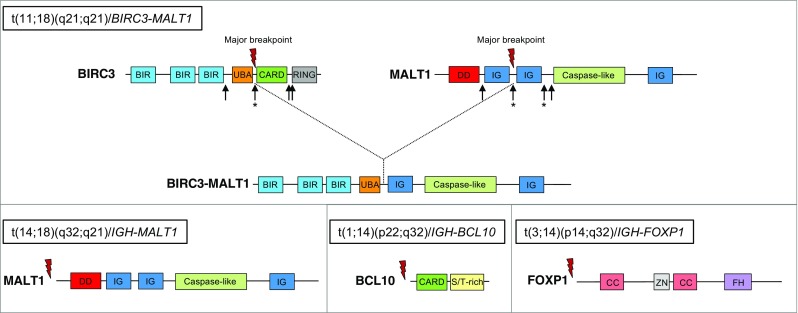
Common translocations in extranodal marginal zone lymphoma. Schematic representation of the proteins BIRC3, MALT1, BCL10, and FOXP1 that are affected by recurrent translocations generating either fusion protein (BIRC3-MALT1) or overexpression of the full-length coding sequence in case of translocations involving the immunoglobulin heavy chain locus (IGH). Arrows indicate the different breakpoints mapped within the BIRC3 and MALT1 gene, respectively. Asterisk marks the most predominant breakpoints. BIR, baculovirus inhibitor of apoptosis repeat; UBA, ubiquitin-associated domain; CARD, caspase recruitment domain; RING, a “really new interesting new gene” domain, encoding a C_3_HC_4_ zinc finger involved in the ubiquitination pathway; DD, death domain; IG, immunoglobulin-like domain; S/T-rich, serine and threonine-rich domain; CC, coiled-coil domain; ZN, zinc finger domain; FH, forkhead domain

There are several recurrent numerical and structural chromosomal aberrations linked to the pathogenesis of EMZL, including trisomy of chromosomes 3, 12, and 18, which are present in 20–30% of the EMZL cases [60–62], and the mutually exclusive chromosomal translocations t(11;18)(q21;q21)/*BIRC3-MALT1*, t(14;18)(q32;q21)/IGH*-MALT1*, t(1;14)(p22;q32)/IGH*-BCL10*, and t(3;14)(p14;q32)/IGH*-FOXP1* [63–68] (Table 1, Fig. 2). The most common is the t(11;18)(q21;q21)/*BIRC3-MALT1* translocation (previously known as *API2-MALT1*), which occurs in approximately 20% of the EMZL cases with a higher predominance at certain sites, such as the lung (45%) and stomach (23%), where it strongly correlates with *H. pylori*-independent variants of gastric EMZL [69, 70]. The *BIRC3-MALT1* translocation is specific for EMZL and has not been detected in SMZL or NMZL. Translocations involving the protease and scaffold protein MALT1 or adaptor protein BCL10 result in activation of the NF-κB pathway [71], while overexpression of transcription factor FOXP1 potentiates WNT/β-catenin signaling and regulates NF-κB activity [72, 73]. Rare translocations of *FOXP1* involving non-IGH partner genes have also been reported, but these may lead to aberrant expression of N-truncated isoforms of FOXP1 [68, 74, 75].

Occasionally, chromosomal translocations and gene amplifications involving transcription factor BCL6 on 3q27.3 have been described in EMZL [76]. Other rare translocations involving IGH in EMZL include t(X;14)(p11;q32)/IGH*-GPR34*[77, 78], t(5;14)(q34;q32)/IGH*-TENM2*, and t(9;14)(p24;q32)/IGH*-KDM4C*[79]. *GPR34* encodes an orphan G protein-coupled receptor highly expressed in immune cells, while TENM2 represents a teneurin transmembrane protein regulating cell-cell contact. KDM4C is one of the JmjC domain-containing histone demethylases involved in epigenetic regulation. Next to these translocations, gains of 6p25 are detected rather exclusively in 20% of the ocular adnexa EMZL cases. Furthermore, deletions on 6q23 of *TNFAIP3* (*A20*), which acts as a negative regulator of the NF-κB pathway, are found across different anatomical sites, but preferentially in translocation-negative EMZL [80–83].

### Somatic mutations

Due to aberrant somatic hypermutation caused by mistargeting of activation-induced cytidine deaminase (AID) in the germinal center reaction, 5′ regulatory regions and coding sequences of proto-oncogenes are mutated in EMZL. Thus, mutations in the 5′ non-coding region of *BCL6* have been identified in 85% of low-grade gastric lymphomas of the EMZL type [84], while somatic missense mutations in *PIM1* and *MYC* have been reported in 30–40% of EMZL (gastric and non-gastric sites) [85, 86]. Gain-of-function mutations in EMZL have also been identified in *BCL10* (6%), *MYD88* (6%), which both lead to NF-κB activation, as well as in *NOTCH1* (8%) and *NOTCH2* (8%) in ocular adnexal EMZL [87–90]. In this same tumor type, inactivating mutations have been found in *TNFAIP3* (27–54%), *TBL1XR1* (18%), *CREBBP* (17%), *TP53* (8%), and *KMTD2* (6–22%) [89, 91].

## Activation of the NF-κB pathway in EMZL

**Fig. 3:**
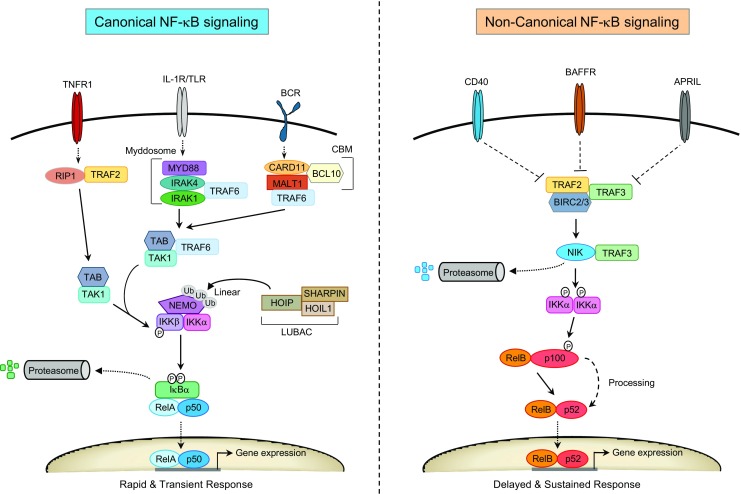
Overview of the canonical and non-canonical NF-κB signaling pathways. Canonical NF-κB signaling is induced upon activation of cytokine receptors (e.g., TNFR1, IL-1R), pattern recognition Toll-like receptors (TLR) or B cell and T cell antigen receptors (BCR or TCR). Each of the different receptor subtypes employ distinct adaptor proteins and signaling complexes (e.g., Myddosome, CBM) that converge and engage the IKK complex, consisting of the regulatory subunit NEMO and the catalytic subunits IKKα and IKKβ. NEMO is regulated in multiple ways including through linear polyubiquitination by the LUBAC complex consisting of HOIP, HOIL1, and SHARPIN. IKK phosphorylation of serine residues on cytosolic IκBs (IκBα/β/ε) or their precursors triggers IκB ubiquitination and proteosomal degradation. Classical NF-κB dimers, like p50/RelA and p50/c-Rel are released and enter the nucleus to regulate gene expression. Under physiological conditions the canonical NF-κB pathway induces rapid but transient transcriptional responses. Non-canonical NF-κB signaling is regulated by kinase NIK, which is normally degraded in resting cells by an E3 ligase complex consisting of TRAF2/TRAF3 adaptor proteins and the E3 ligases BIRC2/3. Activation of a specific subset of TNFR family members, like CD40, BAFFR, or APRIL, leads to stabilization of NIK via inactivation of the TRAF/BIRC complex. Increased NIK protein levels promote IKKα phosphorylation, which in turn phosphorylates RelB/p100, thereby inducing partial proteosomal processing of p100 leading to release of RelB/p52 dimers that translocate to the nucleus. Non-canonical NF-κB signaling results in a more delayed and sustained transcriptional response

NF-κB consists of a family of dimeric transcription factors that are critical for both innate and adaptive immune responses [92]. There are five NF-κB subunits, including RelA (p65), RelB, c-Rel, NF-κB1 (p50 and its precursor p105), and NF-κB2 (p52 and its precursor p100), which are kept inactive in the cytoplasm by their inhibitors (IκBα, IκBβ, and IκBε) or in its dormant precursor form. RelB forms transcriptional inactive complexes with the subunits RelA and c-Rel. NF-κB activation is mediated by two parallel signaling pathways, termed the canonical (classical) and non-canonical (alternative) NF-κB pathway that under normal physiological conditions involves a highly regulated process of transient activation in response to extracellular signals (Fig. 3). The canonical pathway is activated by stimulation of specific receptors, such as the BCR, TLR, and interleukin 1 receptor (IL1R). Each of these receptors engages distinct adaptor molecules, but all converge on the canonical NF-κB pathway, which involves IκB phosphorylation by the IκB kinase (IKK) complex, inducing its K48-linked polyubiquitination and subsequent degradation by the proteasome. As a result, NF-κB homo- and heterodimers are released permitting their translocation to the nucleus and transcriptional regulation of NF-κB target genes. The non-canonical NF-κB pathway consists of successive activation of NF-κB inducible kinase (NIK) and IKKα, leading to phosphorylation and partial proteolysis of NF-κB2 (p100), thereby generating the functional active form p52 that associates with RelB, and upon nuclear translocation regulates transcription [92].

Stimulation of TLR and IL1R triggers dimerization and conformational change of the Toll/IL1R homologous (TIR) domain, which results in recruitment of MYD88, interleukin-1 receptor-associated kinase-4 (IRAK4) and IRAK1, forming the Myddosome complex that is capable of activating the IKK complex and activation of the canonical NF-κB pathway [93]. In-frame deletions and hotspot mutations of *MYD88*, such as p.L265P in the TIR domain, are found in about 19% of the ocular adnexal EMZL cases [89], which yields a gain-of-function phenotype resulting in spontaneous assembly of the Myddosome and activation of NF-κB.

Engagement of the BCR triggers tyrosine phosphorylation of immunoreceptor tyrosine-based activation motif (ITAM) of CD79A and CD79B, which results in recruitment of spleen tyrosine kinase (SYK). Through subsequent activation of Bruton’s tyrosine kinase (BTK) and protein kinase C (PKC) signaling, the scaffold protein CARD11 (CARMA1) is recruited, which upon a conformational change is able to interact with adaptor protein BCL10, thereby promoting its polymerization and filament formation leading to assembly of the CARD11/BCL10/MALT1 (CBM) signalosome complex [94]. The CBM complex recruits then TNFR-associated factor-6 (TRAF6), transforming growth factor β activating kinase-1 (TAK1) and TAK binding protein-2/3 (TAB2/3), which leads to activation of the IKK complex and stimulation of the canonical NF-κB signaling pathway [95]. Overexpression of BCL10 due to t(1;14)(p22;q32) causes its constitutive activation through oligomerization via its N-terminal caspase recruitment domain (CARD)/CARD interaction, thus leading to enhanced NF-κB signaling. BCL10 also regulates the non-canonical NF-κB pathway, which normally acts downstream of receptors, like CD40 and B cell activating factor receptor (BAFFR).

**Fig. 4:**
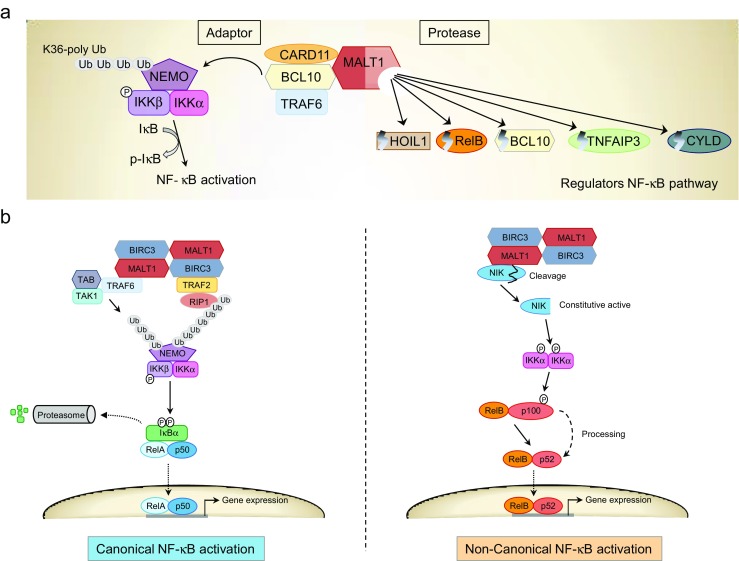
Regulation of the NF-κB signaling pathway by MALT1 and BIRC3-MALT1. **a** Overview of MALT1-dependent activation and regulation of the NF-κB pathway via its adaptor and protease functions. Through its adaptor functions, it recruits the CARD11/BCL10/TRAF6 complex, which results in polyubiquitination and degradation of NEMO, thereby activating the IKK complex, which phosphorylates IκB and activates NF-κB. MALT1 protease activity controls NF-κB activation by promoting the degradation of both positive (HOIL1, RelB, BCL10) and negative (TNFAIP3, CYLD) regulators of this pathway. **b** The chimeric fusion protein BIRC3-MALT1 is created by the chromosomal translocation t(11;18)(q21;q21) in extranodal marginal zone lymphoma. The oncogenic potential of BIRC3-MALT1 relies on its ability to activate both the canonical and non-canonical pathways through multiple mechanisms. BIRC3-MALT1 is activated through auto-oligomerization, which results in the recruitment of TRAF2/RIP1 via the BIRC3 moiety that triggers RIP1 ubiquitination and canonical NF-κB activation. In addition, recruitment of TRAF6/TAB/TAK1 induces NEMO ubiquitination and also canonical NF-κB signaling. In parallel, BIRC3-MALT1 causes deregulated MALT1 paracaspase activity, which results in proteolytic cleavage of NIK, creating a constitutively active NIK fragment that stimulates IKKα and the non-canonical NF-κB pathway

The paracaspase MALT1 is an Arg-specific protease that contains several functional domains including an N-terminal death domain, three immunoglobulin (Ig)-like domains and a proteolytically active caspase-like domain [96]. As a result of t(14;18)(q32;q21), increased levels of MALT1 facilitate the interaction with BCL10 through its N-terminal Ig-like domains, which triggers its own oligomerization and activation, thus enhancing canonical NF-κB signaling (Fig. 4a). Furthermore, through its protease activity, MALT1 also promotes the specific cleavage of several negative regulators of NF-κB, which includes TNFAIP3, BCL10, CYLD, and RelB [97] (Fig. 4a). TNFAIP3 can inactivate a number of NF-κB signaling molecules, like receptor-interacting protein 1/2 (RIP1/2), TRAFF6, and IKKγ (NEMO). Thus, *TNFAIP3* deletions and inactivating mutations, which are predominantly observed in translocation-negative EMZL of ocular adnexa (30%), salivary glands (8%), and thyroid (11%), augment NF-κB signaling downstream of multiple surface receptors [82, 98].

Recently, another substrate of MALT1 has been identified that is also linked to regulation of the NF-κB pathway [99, 100]. This involves HOIL1(RBCK1), a component of the linear ubiquitin chain assembly complex (LUBAC), which comprises of HOIL1, HOIP(RNF31), and Sharpin. LUBAC promotes NF-κB activation by addition of linear (N-terminal linked) polyubiquitin chains on its substrates. MALT1-dependent RBCK1 cleavage reduces linear ubiquitination of cellular proteins and has thus been proposed to provide negative feedback on the NF-κB pathway.

The BIRC3-MALT1 fusion protein resulting from t(11;18)(q21;q21) gains novel functions through its ability to constitutively activate both canonical and non-canonical NF-κB pathways [101] (Fig. 4b). BIRC3 belongs to the inhibitor of apoptosis (IAP) family of proteins and contains three tandem copies of the baculovirus IAP repeat (BIR) domain, a CARD and a C-terminal RING domain. Several variants of the BIRC3-MALT1 fusion are present in patients with t(11;18)(q21;q21) translocation [65, 102, 103]. In all cases, the breakpoints within *BIRC3* occur consistently between the third BIR and the CARD domain, whereas the breakpoints within *MALT1* retain the C-terminal caspase-like domain. The BIRC3-MALT1 fusion is capable of auto-oligomerization, recruitment of TRAF2/RIP1 and TRAF6/TAB/TAK1 complexes, as well as cleavage of TNFAIP3 and CYLD, thereby activating the canonical NF-κB pathway. In addition, the BIRC3 moiety of the fusion protein recruits NIK, leading to its cleavage by the MALT protease domain [104]. The resulting truncated NIK kinase domain is resistant to TRAF3-dependent proteosomal degradation, leading to constitutive activation of the non-canonical NF-κB pathway. Finally, the BIRC3-MALT1 fusion protein has also the ability to cleave the tumor suppressor protein LIM domain and actin-binding protein-1 (LIMA1), thereby generating a novel oncogenic LIM domain only (LMO) fragment [105].

## Progression and histological transformation of EMZL

EMZL is normally presented as a low-grade tumor, but in some cases gradually develops into a more aggressive large B cell lymphoma with often complete transformation into DLBCL. During this transition, composite lymphomas may exist showing fields of clonally related small and large cell areas. Histological transformation to DLBCL has been observed between 3 and 4% [106, 107] and 8–11% of the EMZL cases [5, 108]. Although there is a stronger tendency of t(11;18)-negative EMZL to transform into DLBCL [109, 110], the presence of *BIRC3-MALT* translocation in gastric EMZL does not exclude progression to DLBCL [111, 112]. Progression of low-grade lymphoma toward high-grade lymphoma is facilitated by complete loss of *p16INK4A* and *TP53* gene function [113, 114]. Furthermore, chromosomal translocations involving *BCL6*[115–117], or *CCND3*[118], as well as MYC overexpression [119], and strong nuclear FOXP1 expression [120] are found in DLBCL transformation. In addition, upregulation of the chemokine receptors CXCR3 and CXCR7 has been correlated with progression of gastric EMZL into DLBCL [121].

## Diagnosis of EMZL

The diagnosis of EMZL can be rather challenging, as extranodal sites of disease are sometimes difficult to access, resulting in small biopsy samples. The optimal diagnosis of EMZL requires integration of clinical, histopathological, and molecular information.

### Histopathological findings

In many cases, EMZL consists of multifocal, small, or confluent, clonally identical foci of malignant cells that colonize the germinal center and are scattered throughout the involved organ. EMZL shows a morphological spectrum, ranging from mixtures of heterogeneous B cells, including monocytoid and plasmacytoid B cells, small lymphocytes, and centrocytes to monomorphic proliferations of monocytoid B cells. In about one-third of the cases, prominent plasmacytic differentiation is observed. Besides the tumor cells, additional reactive cells are present, consisting mainly of T lymphocytes. Other histological features include remnants of reactive follicular hyperplasia and infiltration of glands or crypts of adjacent tissue accompanied by architectural destruction, resulting in lymphoepithelial lesions (LEL). The EMZL cells are positive for CD20, CD22, CD35, CD79a, BCL2, and IgM, while usually negative for CD5, CD10, CD23, cyclin D1, BCL6, and IgD, and many of these markers are informative for differential diagnosis. Both flow cytometry and immunohistochemistry can be performed to detect the expression of these markers. Additional immunohistochemical markers that are informative include MNDA and IRTA1 [122–125], as well as the detection of MALT1 and BCL10 nuclear/cytoplasmic protein levels in 18q21 and 1p22 translocation-positive EMZL [64, 126, 127].

### Molecular diagnostics

#### IG clonality testing

Although histopathological examination remains the gold standard for diagnosis, the detection of monoclonality of immunoglobulin (IG) gene rearrangements, preferably using the EuroClonality/BIOMED-2 primer sets and protocols, represents a useful aid [128]. Especially, inclusion of incomplete IGH-DJ joining as a clonality target is very informative, since clonal IGH-DJ rearrangements occur in many EMZL cases. Furthermore, clonal IGH-DJ rearrangements are exclusively present in 5–8% of clonal B cell populations in the absence of detectable IGH-VJ rearrangements [129]. Although not part of the routine diagnostic workup, sequence analysis of the rearranged IGHV genes in EMZL have further provided evidence for antigen mediated affinity maturation by the restricted use of certain sequences. In extranodal lymphomas located at the ocular adnexa and salivary glands there is biased usage of IGH4-34 and IGHV1-69, respectively, while those in the stomach appear to have overrepresentation of IGHV3-7 and IGHV1-69 usage [130–132].

#### Detection of chromosomal aberrations by FISH and RT-PCR

The detection of common cytogenetic abnormalities by interphase fluorescence in situ hybridization (FISH) has been proven to be informative for the diagnosis of EMZL [133, 134]. FISH is used for the detection of chromosomal translocations involving IGH*, MALT1*, *FOXP1*, and *BCL10*, as well as numerical chromosomal abnormalities, including deletions and trisomy of chromosome 3, 12, and 18 [59, 133, 135, 136]. However, it should be emphasized, that while positive FISH together with clinical and morphological features of EMZL is very helpful in the diagnosis, negative FISH should not exclude the diagnosis of EMZL. Through the identification of the specific genomic regions rearranged in EMZL, routine reverse transcription polymerase chain reaction (RT-PCR) has also been implemented for the detection of genomic translocations and the presence of fusion transcripts, such as *BIRC3-MALT1*[137, 138]. Moreover, detection of *BIRC3-MALT1* in gastric EMZL has therapeutic implications (see below).

## Therapeutic strategies for EMZL

The involvement of infectious agents in the pathogenesis of EMZL has provided opportunities toward unique therapeutic approaches for lymphoma treatment. Many patients with ocular adnexa EMZL respond to doxycycline treatment and show lymphoma regression in 65% of the patients [139]. Likewise, for stages I and II of gastric EMZL, the initial treatment of choice is *H. pylori* eradication, which results in complete remission in about 80% of patients with gastric EMZL [140–142]. The most commonly used regimen includes a proton pump inhibitor (omeprazole) in combination with amoxicillin and clarithromycin. Notably, EMZL harboring t(11;18) and t(1;14) translocations are associated with resistance to *H. pylori* eradication therapy [70, 141]. *H. pylori*-negative patients also respond to antibiotic treatment, since other microorganisms are known to be involved in the pathogenesis of gastric EMZL, and complete remission can be achieved in 57% of these patients [143]. Patients with symptomatic systemic disease, mainly those with disseminated stages III and IV, are considered for treatment with chemotherapy (e.g., bendamustine, fludarabine, or chlorambucil) combined with either anti-CD20 antibody rituximab or the immunomodulatory drug lenalidomide [144–146]. First-line treatment combining chlorambucil with rituximab has shown improved survival as compared to chlorambucil or rituximab alone [146]. Combination therapy of rituximab with lenalidomide has also been demonstrated to be effective [147], along with cyclophosphamide and dexamethasone [148]. The more aggressive types of chemotherapy regimens, including CHOP (cyclophosphamide, doxorubicine, vincristine, and prednisone), are often reserved for patients with transformation to high-grade lymphomas.

**Fig. 5:**
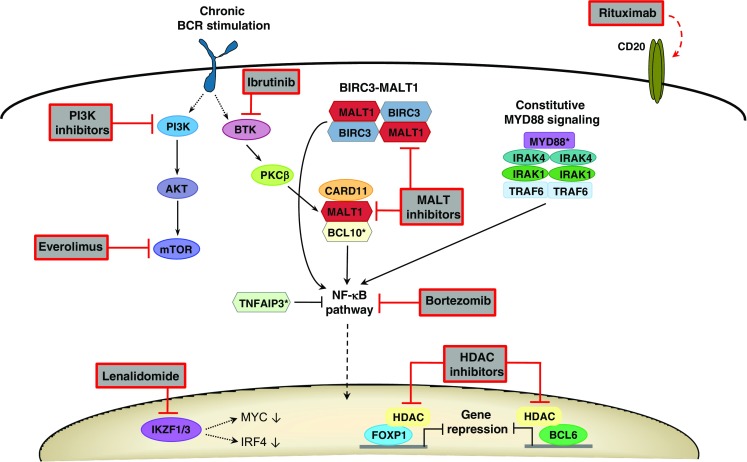
Targeted therapy in extranodal marginal zone lymphoma. Rituximab targets CD20-positive B lymphoma cells, which results in anti-tumor effects related to complement-dependent cellular cytotoxicity and antibody-dependent cellular cytotoxicity. Upstream of the NF-κB pathway, different types of inhibitors may target key enzymes that are activated downstream of BCR signaling or affected by genetic alterations (*BIRC3-MALT1* translocation, *BCL10* and *MYD88* mutations [*]). These include BTK inhibitor inbrutinib and MALT inhibitors. BCR stimulation also activates the PI3K/AKT/mTOR pathway that can be targeted by PI3K inhibitors and mTOR inhibitor everolimus. Activation of NF-κB itself, which may also result by loss of TNFAIP3 function due to gene deletions or mutations [*], can be blocked by bortezomib. Lenalidomide promotes degradation of IKZF1 and IKZF3, thereby downregulating the expression of MYC and IRF4. Transcription factors FOXP1 and BCL6, which are upregulated by genetic alterations (e.g., chromosomal translocations, promoter mutations), as well as other epigenetic regulators that require HDAC activity, can be blocked by the action of HDAC inhibitors

Alternative therapies for EMZL involving new agents include inhibitors of mTOR (everolimus) [149], HDAC (vorinostat) [150, 151], proteasome (bortezomib) [152], BTK (ibrutinib) [153, 154], and PI3Kδ (idelalisib) [155] (Fig. 5). Many of these drugs are under investigation in clinical trials, of which some show positive response rates, but improvement on long-term overall survival remains to be demonstrated. Targeted therapy directed against the MALT1 paracaspase protein has also been exploited for therapeutic intervention. Several inhibitors have been identified that show promising results in activated B cell-DLBCL [156–158], but their effectiveness in EMZL remains to be established.

## Conclusions

During the past two decades, new insight has been gained into the pathobiology of EMZL, which revealed a complex interplay between chronic inflammation and genetic abnormalities that seem to converge on deregulation of specific signaling cascades that often result in activation of the NF-κB pathway. This knowledge has lead to new developments in clinical diagnostics and has opened interesting opportunities for more targeted therapeutic intervention. Further understanding of which specific molecules within these signaling pathways are essential in promoting and maintaining lymphomagenesis may lead to novel therapy modalities, which will be especially relevant for managing the more aggressive forms of EMZL.

## References

[1] Thieblemont C (2005) Clinical presentation and management of marginal zone lymphomas. Hematology Am Soc Hematol Educ Program 307–313. 10.1182/asheducation-2005.1.307 10.1182/asheducation-2005.1.307 16304396

[2] Du MQ (2016). MALT lymphoma: a paradigm of NF-kappaB dysregulation. Semin Cancer Biol.

[3] Zucca E, Bertoni F, Vannata B, Cavalli F (2014). Emerging role of infectious etiologies in the pathogenesis of marginal zone B-cell lymphomas. Clin Cancer Res: Off J Am Assoc Cancer Res.

[4] Olszewski AJ, Castillo JJ (2013). Survival of patients with marginal zone lymphoma: analysis of the surveillance, epidemiology, and end results database. Cancer.

[5] Meyer AH, Stroux A, Lerch K, Eucker J, Eitle J, Hohloch K, Andrzejak M, Possinger K, Dorken B, Pezzutto A, Scholz CW (2014). Transformation and additional malignancies are leading risk factors for an adverse course of disease in marginal zone lymphoma. Ann Oncol: Off J Eur Soc Med Oncol/ESMO.

[6] Sretenovic M, Colovic M, Jankovic G, Suvajdzic N, Mihaljevic B, Colovic N, Todorovic M, Atkinson HD (2009). More than a third of non-gastric malt lymphomas are disseminated at diagnosis: a single center survey. Eur J Haematol.

[7] Thieblemont C, Berger F, Dumontet C, Moullet I, Bouafia F, Felman P, Salles G, Coiffier B (2000). Mucosa-associated lymphoid tissue lymphoma is a disseminated disease in one third of 158 patients analyzed. Blood.

[8] Bertoni F, Cazzaniga G, Bosshard G, Roggero E, Barbazza R, De Boni M, Capella C, Pedrinis E, Cavalli F, Biondi A, Zucca E (1997). Immunoglobulin heavy chain diversity genes rearrangement pattern indicates that MALT-type gastric lymphoma B cells have undergone an antigen selection process. Br J Haematol.

[9] Craig VJ, Arnold I, Gerke C, Huynh MQ, Wundisch T, Neubauer A, Renner C, Falkow S, Muller A (2010). Gastric MALT lymphoma B cells express polyreactive, somatically mutated immunoglobulins. Blood.

[10] Wohrer S, Troch M, Streubel B, Zwerina J, Skrabs C, Formanek M, Hauff W, Hoffmann M, Mullauer L, Chott A, Raderer M (2007). MALT lymphoma in patients with autoimmune diseases: a comparative analysis of characteristics and clinical course. Leukemia.

[11] Ekstrom Smedby K, Vajdic CM, Falster M, Engels EA, Martinez-Maza O, Turner J, Hjalgrim H, Vineis P, Seniori Costantini A, Bracci PM, Holly EA, Willett E, Spinelli JJ, La Vecchia C, Zheng T, Becker N, De Sanjose S, Chiu BC, Dal Maso L, Cocco P, Maynadie M, Foretova L, Staines A, Brennan P, Davis S, Severson R, Cerhan JR, Breen EC, Birmann B, Grulich AE, Cozen W (2008). Autoimmune disorders and risk of non-Hodgkin lymphoma subtypes: a pooled analysis within the InterLymph Consortium. Blood.

[12] Luminari S, Cesaretti M, Marcheselli L, Rashid I, Madrigali S, Maiorana A, Federico M (2010). Decreasing incidence of gastric MALT lymphomas in the era of anti-Helicobacter pylori interventions: results from a population-based study on extranodal marginal zone lymphomas. Ann Oncol: Off J Eur Soc Med Oncol/ESMO.

[13] Hussell T, Isaacson PG, Crabtree JE, Spencer J (1993). The response of cells from low-grade B-cell gastric lymphomas of mucosa-associated lymphoid tissue to Helicobacter pylori. Lancet.

[14] Munari F, Lonardi S, Cassatella MA, Doglioni C, Cangi MG, Amedei A, Facchetti F, Eishi Y, Rugge M, Fassan M, de Bernard M, D'Elios MM, Vermi W (2011). Tumor-associated macrophages as major source of APRIL in gastric MALT lymphoma. Blood.

[15] Wang HP, Zhu YL, Shao W (2013). Role of Helicobacter pylori virulence factor cytotoxin-associated gene A in gastric mucosa-associated lymphoid tissue lymphoma. World J Gastroenterol.

[16] Tohidpour A (2016). CagA-mediated pathogenesis of Helicobacter pylori. Microb Pathog.

[17] Lin WC, Tsai HF, Kuo SH, Wu MS, Lin CW, Hsu PI, Cheng AL, Hsu PN (2010). Translocation of Helicobacter pylori CagA into human B lymphocytes, the origin of mucosa-associated lymphoid tissue lymphoma. Cancer Res.

[18] Selbach M, Moese S, Hauck CR, Meyer TF, Backert S (2002). Src is the kinase of the Helicobacter pylori CagA protein in vitro and in vivo. J Biol Chem.

[19] Poppe M, Feller SM, Romer G, Wessler S (2007). Phosphorylation of Helicobacter pylori CagA by c-Abl leads to cell motility. Oncogene.

[20] Higashi H, Tsutsumi R, Muto S, Sugiyama T, Azuma T, Asaka M, Hatakeyama M (2002). SHP-2 tyrosine phosphatase as an intracellular target of Helicobacter pylori CagA protein. Science.

[21] Zhu Y, Wang C, Huang J, Ge Z, Dong Q, Zhong X, Su Y, Zheng S (2007). The Helicobacter pylori virulence factor CagA promotes Erk1/2-mediated bad phosphorylation in lymphocytes: a mechanism of CagA-inhibited lymphocyte apoptosis. Cell Microbiol.

[22] Kuo SH, Chen LT, Lin CW, Yeh KH, Shun CT, Tzeng YS, Liou JM, Wu MS, Hsu PN, Cheng AL (2016) Expressions of the CagA protein and CagA-signaling molecules predict H. pylori-dependence of early-stage gastric DLBCL. Blood. 10.1182/blood-2016-04-713719 10.1182/blood-2016-04-713719 27864293

[23] Buti L, Spooner E, Van der Veen AG, Rappuoli R, Covacci A, Ploegh HL (2011). Helicobacter pylori cytotoxin-associated gene A (CagA) subverts the apoptosis-stimulating protein of p53 (ASPP2) tumor suppressor pathway of the host. Proc Natl Acad Sci U S A.

[24] Umehara S, Higashi H, Ohnishi N, Asaka M, Hatakeyama M (2003). Effects of Helicobacter pylori CagA protein on the growth and survival of B lymphocytes, the origin of MALT lymphoma. Oncogene.

[25] Ohmae T, Hirata Y, Maeda S, Shibata W, Yanai A, Ogura K, Yoshida H, Kawabe T, Omata M (2005). Helicobacter pylori activates NF-kappaB via the alternative pathway in B lymphocytes. J Immunol.

[26] Haesebrouck F, Pasmans F, Flahou B, Smet A, Vandamme P, Ducatelle R (2011). Non-Helicobacter pylori Helicobacter species in the human gastric mucosa: a proposal to introduce the terms H. heilmannii sensu lato and sensu stricto. Helicobacter.

[27] Bento-Miranda M, Figueiredo C (2014). Helicobacter heilmannii sensu lato: an overview of the infection in humans. World J Gastroenterol.

[28] Stolte M, Bayerdorffer E, Morgner A, Alpen B, Wundisch T, Thiede C, Neubauer A (2002). Helicobacter and gastric MALT lymphoma. Gut.

[29] Ferreri AJ, Guidoboni M, Ponzoni M, De Conciliis C, Dell’Oro S, Fleischhauer K, Caggiari L, Lettini AA, Dal Cin E, Ieri R, Freschi M, Villa E, Boiocchi M, Dolcetti R (2004). Evidence for an association between Chlamydia psittaci and ocular adnexal lymphomas. J Natl Cancer Inst.

[30] Ferreri AJ, Ponzoni M, Guidoboni M, Resti AG, Politi LS, Cortelazzo S, Demeter J, Zallio F, Palmas A, Muti G, Dognini GP, Pasini E, Lettini AA, Sacchetti F, De Conciliis C, Doglioni C, Dolcetti R (2006). Bacteria-eradicating therapy with doxycycline in ocular adnexal MALT lymphoma: a multicenter prospective trial. J Natl Cancer Inst.

[31] Chanudet E, Zhou Y, Bacon CM, Wotherspoon AC, Muller-Hermelink HK, Adam P, Dong HY, de Jong D, Li Y, Wei R, Gong X, Wu Q, Ranaldi R, Goteri G, Pileri SA, Ye H, Hamoudi RA, Liu H, Radford J, Du MQ (2006). Chlamydia psittaci is variably associated with ocular adnexal MALT lymphoma in different geographical regions. J Pathol.

[32] Daibata M, Nemoto Y, Togitani K, Fukushima A, Ueno H, Ouchi K, Fukushi H, Imai S, Taguchi H (2006). Absence of Chlamydia psittaci in ocular adnexal lymphoma from Japanese patients. Br J Haematol.

[33] Rosado MF, Byrne GE, Ding F, Fields KA, Ruiz P, Dubovy SR, Walker GR, Markoe A, Lossos IS (2006). Ocular adnexal lymphoma: a clinicopathologic study of a large cohort of patients with no evidence for an association with Chlamydia psittaci. Blood.

[34] Husain A, Roberts D, Pro B, McLaughlin P, Esmaeli B (2007). Meta-analyses of the association between Chlamydia psittaci and ocular adnexal lymphoma and the response of ocular adnexal lymphoma to antibiotics. Cancer.

[35] Ponzoni M, Ferreri AJ, Guidoboni M, Lettini AA, Cangi MG, Pasini E, Sacchi L, Pecciarini L, Grassi S, Dal Cin E, Stefano R, Magnino S, Dolcetti R, Doglioni C (2008). Chlamydia infection and lymphomas: association beyond ocular adnexal lymphomas highlighted by multiple detection methods. Clin Cancer Res: Off J Am Assoc Cancer Res.

[36] Dingle KE, Van Den Braak N, Colles FM, Price LJ, Woodward DL, Rodgers FG, Endtz HP, Van Belkum A, Maiden MC (2001). Sequence typing confirms that Campylobacter jejuni strains associated with Guillain-Barre and Miller-Fisher syndromes are of diverse genetic lineage, serotype, and flagella type. J Clin Microbiol.

[37] Lecuit M, Abachin E, Martin A, Poyart C, Pochart P, Suarez F, Bengoufa D, Feuillard J, Lavergne A, Gordon JI, Berche P, Guillevin L, Lortholary O (2004). Immunoproliferative small intestinal disease associated with Campylobacter jejuni. N Engl J Med.

[38] Ben-Ayed F, Halphen M, Najjar T, Boussene H, Jaafoura H, Bouguerra A, Ben Salah N, Mourali N, Ayed K, Ben Khalifa H (1989). Treatment of alpha chain disease. Results of a prospective study in 21 Tunisian patients by the Tunisian-French Intestinal Lymphoma Study Group. Cancer.

[39] Goodlad JR, Davidson MM, Hollowood K, Ling C, MacKenzie C, Christie I, Batstone PJ, Ho-Yen DO (2000). Primary cutaneous B-cell lymphoma and Borrelia burgdorferi infection in patients from the highlands of Scotland. Am J Surg Pathol.

[40] Cerroni L, Zochling N, Putz B, Kerl H (1997). Infection by Borrelia burgdorferi and cutaneous B-cell lymphoma. J Cutan Pathol.

[41] Ponzoni M, Ferreri AJ, Mappa S, Pasini E, Govi S, Facchetti F, Fanoni D, Tucci A, Vino A, Doglioni C, Berti E, Dolcetti R (2011). Prevalence of Borrelia burgdorferi infection in a series of 98 primary cutaneous lymphomas. Oncologist.

[42] de la Fouchardiere A, Vandenesch F, Berger F (2003). Borrelia-associated primary cutaneous MALT lymphoma in a nonendemic region. Am J Surg Pathol.

[43] Adam P, Czapiewski P, Colak S, Kosmidis P, Tousseyn T, Sagaert X, Boudova L, Okon K, Morresi-Hauf A, Agostinelli C, Pileri S, Pruneri G, Martinelli G, Du MQ, Fend F (2014). Prevalence of Achromobacter xylosoxidans in pulmonary mucosa-associated lymphoid tissue lymphoma in different regions of Europe. Br J Haematol.

[44] Luppi M, Longo G, Ferrari MG, Ferrara L, Marasca R, Barozzi P, Morselli M, Emilia G, Torelli G (1996). Additional neoplasms and HCV infection in low-grade lymphoma of MALT type. Br J Haematol.

[45] Michot JM, Canioni D, Driss H, Alric L, Cacoub P, Suarez F, Sibon D, Thieblemont C, Dupuis J, Terrier B, Feray C, Tilly H, Pol S, Leblond V, Settegrana C, Rabiega P, Barthe Y, Hendel-Chavez H, Nguyen-Khac F, Merle-Beral H, Berger F, Molina T, Charlotte F, Carrat F, Davi F, Hermine O, Besson C, Group AH-L-CS (2015). Antiviral therapy is associated with a better survival in patients with hepatitis C virus and B-cell non-Hodgkin lymphomas, ANRS HC-13 lympho-C study. Am J Hematol.

[46] Marcucci F, Mele A (2011). Hepatitis viruses and non-Hodgkin lymphoma: epidemiology, mechanisms of tumorigenesis, and therapeutic opportunities. Blood.

[47] Skopouli FN, Dafni U, Ioannidis JP, Moutsopoulos HM (2000). Clinical evolution, and morbidity and mortality of primary Sjogren’s syndrome. Semin Arthritis Rheum.

[48] Papageorgiou A, Voulgarelis M, Tzioufas AG (2015). Clinical picture, outcome and predictive factors of lymphoma in Sjgren syndrome. Autoimmun Rev.

[49] Ambrosetti A, Zanotti R, Pattaro C, Lenzi L, Chilosi M, Caramaschi P, Arcaini L, Pasini F, Biasi D, Orlandi E, D'Adda M, Lucioni M, Pizzolo G (2004). Most cases of primary salivary mucosa-associated lymphoid tissue lymphoma are associated either with Sjoegren syndrome or hepatitis C virus infection. Br J Haematol.

[50] Brito-Zeron P, Kostov B, Fraile G, Caravia-Duran D, Maure B, Rascon FJ, Zamora M, Casanovas A, Lopez-Dupla M, Ripoll M, Pinilla B, Fonseca E, Akasbi M, de la Red G, Duarte-Millan MA, Fanlo P, Guisado-Vasco P, Perez-Alvarez R, Chamorro AJ, Morcillo C, Jimenez-Heredia I, Sanchez-Berna I, Lopez-Guillermo A, Ramos-Casals M, GEAS-SEMI SSSG (2017). Characterization and risk estimate of cancer in patients with primary Sjogren syndrome. J Hematol Oncol.

[51] Streubel B, Huber D, Wohrer S, Chott A, Raderer M (2004). Frequency of chromosomal aberrations involving MALT1 in mucosa-associated lymphoid tissue lymphoma in patients with Sjogren’s syndrome. Clin Cancer R: Off J Am Assoc Cancer Res.

[52] Papageorgiou A, Mavragani CP, Nezos A, Zintzaras E, Quartuccio L, De Vita S, Koutsilieris M, Tzioufas AG, Moutsopoulos HM, Voulgarelis M (2015). A BAFF receptor His159Tyr mutation in Sjogren’s syndrome-related lymphoproliferation. Arthritis Rheumatol.

[53] Nocturne G, Boudaoud S, Miceli-Richard C, Viengchareun S, Lazure T, Nititham J, Taylor KE, Ma A, Busato F, Melki J, Lessard CJ, Sivils KL, Dubost JJ, Hachulla E, Gottenberg JE, Lombes M, Tost J, Criswell LA, Mariette X (2013). Germline and somatic genetic variations of TNFAIP3 in lymphoma complicating primary Sjogren's syndrome. Blood.

[54] Ellis GL (2007). Lymphoid lesions of salivary glands: malignant and benign. Med Oral Patol Oral Cir Bucal.

[55] Carbone A, Gloghini A, Ferlito A (2000). Pathological features of lymphoid proliferations of the salivary glands: lymphoepithelial sialadenitis versus low-grade B-cell lymphoma of the malt type. Ann Otology Rhinol Laryngol.

[56] Pedersen RK, Pedersen NT (1996). Primary non-Hodgkin’s lymphoma of the thyroid gland: a population based study. Histopathology.

[57] Ahmed R, Al-Shaikh S, Akhtar M (2012). Hashimoto thyroiditis: a century later. Adv Anat Pathol.

[58] Chistiakov DA (2005). Immunogenetics of Hashimoto’s thyroiditis. J Autoimmune Dis.

[59] Streubel B, Simonitsch-Klupp I, Mullauer L, Lamprecht A, Huber D, Siebert R, Stolte M, Trautinger F, Lukas J, Puspok A, Formanek M, Assanasen T, Muller-Hermelink HK, Cerroni L, Raderer M, Chott A (2004). Variable frequencies of MALT lymphoma-associated genetic aberrations in MALT lymphomas of different sites. Leukemia.

[60] Brynes RK, Almaguer PD, Leathery KE, McCourty A, Arber DA, Medeiros LJ, Nathwani BN (1996). Numerical cytogenetic abnormalities of chromosomes 3, 7, and 12 in marginal zone B-cell lymphomas. Mod Pathol.

[61] Wotherspoon AC, Finn TM, Isaacson PG (1995). Trisomy 3 in low-grade B-cell lymphomas of mucosa-associated lymphoid tissue. Blood.

[62] Hoeve MA, Gisbertz IA, Schouten HC, Schuuring E, Bot FJ, Hermans J, Hopman A, Kluin PM, Arends JW, van Krieken JH (1999). Gastric low-grade MALT lymphoma, high-grade MALT lymphoma and diffuse large B cell lymphoma show different frequencies of trisomy. Leukemia.

[63] Willis TG, Jadayel DM, Du MQ, Peng H, Perry AR, Abdul-Rauf M, Price H, Karran L, Majekodunmi O, Wlodarska I, Pan L, Crook T, Hamoudi R, Isaacson PG, Dyer MJ (1999). Bcl10 is involved in t(1;14)(p22;q32) of MALT B cell lymphoma and mutated in multiple tumor types. Cell.

[64] Streubel B, Lamprecht A, Dierlamm J, Cerroni L, Stolte M, Ott G, Raderer M, Chott A (2003). T(14;18)(q32;q21) involving IGH and MALT1 is a frequent chromosomal aberration in MALT lymphoma. Blood.

[65] Auer IA, Gascoyne RD, Connors JM, Cotter FE, Greiner TC, Sanger WG, Horsman DE (1997). t(11;18)(q21;q21) is the most common translocation in MALT lymphomas. Ann Oncol.

[66] Ott G, Katzenberger T, Greiner A, Kalla J, Rosenwald A, Heinrich U, Ott MM, Muller-Hermelink HK (1997). The t(11;18)(q21;q21) chromosome translocation is a frequent and specific aberration in low-grade but not high-grade malignant non-Hodgkin’s lymphomas of the mucosa-associated lymphoid tissue (MALT-) type. Cancer Res.

[67] Streubel B, Vinatzer U, Lamprecht A, Raderer M, Chott A (2005). T(3;14)(p14.1;q32) involving IGH and FOXP1 is a novel recurrent chromosomal aberration in MALT lymphoma. Leukemia.

[68] Wlodarska I, Veyt E, De Paepe P, Vandenberghe P, Nooijen P, Theate I, Michaux L, Sagaert X, Marynen P, Hagemeijer A, De Wolf-Peeters C (2005). FOXP1, a gene highly expressed in a subset of diffuse large B-cell lymphoma, is recurrently targeted by genomic aberrations. Leukemia.

[69] Liu H, Ye H, Ruskone-Fourmestraux A, De Jong D, Pileri S, Thiede C, Lavergne A, Boot H, Caletti G, Wundisch T, Molina T, Taal BG, Elena S, Thomas T, Zinzani PL, Neubauer A, Stolte M, Hamoudi RA, Dogan A, Isaacson PG, Du MQ (2002). T(11;18) is a marker for all stage gastric MALT lymphomas that will not respond to H. pylori eradication. Gastroenterology.

[70] Liu H, Ruskon-Fourmestraux A, Lavergne-Slove A, Ye H, Molina T, Bouhnik Y, Hamoudi RA, Diss TC, Dogan A, Megraud F, Rambaud JC, Du MQ, Isaacson PG (2001). Resistance of t(11;18) positive gastric mucosa-associated lymphoid tissue lymphoma to Helicobacter pylori eradication therapy. Lancet.

[71] Du MQ (2011). MALT lymphoma: many roads lead to nuclear factor-kappab activation. Histopathology.

[72] van Keimpema M, Gruneberg LJ, Mokry M, van Boxtel R, Koster J, Coffer PJ, Pals ST, Spaargaren M (2014). FOXP1 directly represses transcription of proapoptotic genes and cooperates with NF-kappaB to promote survival of human B cells. Blood.

[73] Green MR, Gandhi MK, Courtney MJ, Marlton P, Griffiths L (2009). Relative abundance of full-length and truncated FOXP1 isoforms is associated with differential NFkappaB activity in follicular lymphoma. Leuk Res.

[74] Goatly A, Bacon CM, Nakamura S, Ye H, Kim I, Brown PJ, Ruskone-Fourmestraux A, Cervera P, Streubel B, Banham AH, Du MQ (2008). FOXP1 abnormalities in lymphoma: translocation breakpoint mapping reveals insights into deregulated transcriptional control. Mod Pathol.

[75] Rouhigharabaei L, Finalet Ferreiro J, Tousseyn T, van der Krogt JA, Put N, Haralambieva E, Graux C, Maes B, Vicente C, Vandenberghe P, Cools J, Wlodarska I (2014). Non-IG aberrations of FOXP1 in B-cell malignancies lead to an aberrant expression of N-truncated isoforms of FOXP1. PLoS One.

[76] Ye H, Remstein ED, Bacon CM, Nicholson AG, Dogan A, Du MQ (2008). Chromosomal translocations involving BCL6 in MALT lymphoma. Haematologica.

[77] Ansell SM, Akasaka T, McPhail E, Manske M, Braggio E, Price-Troska T, Ziesmer S, Secreto F, Fonseca R, Gupta M, Law M, Witzig TE, Dyer MJ, Dogan A, Cerhan JR, Novak AJ (2012). t(X;14)(p11;q32) in MALT lymphoma involving GPR34 reveals a role for GPR34 in tumor cell growth. Blood.

[78] Baens M, Finalet Ferreiro J, Tousseyn T, Urbankova H, Michaux L, de Leval L, Dierickx D, Wolter P, Sagaert X, Vandenberghe P, De Wolf-Peeters C, Wlodarska I (2012). t(X;14)(p11.4;q32.33) is recurrent in marginal zone lymphoma and up-regulates GPR34. Haematologica.

[79] Vinatzer U, Gollinger M, Mullauer L, Raderer M, Chott A, Streubel B (2008). Mucosa-associated lymphoid tissue lymphoma: novel translocations including rearrangements of ODZ2, JMJD2C, and CNN3. Clin Cancer Res: Off J Am Assoc Cancer Res.

[80] Kim WS, Honma K, Karnan S, Tagawa H, Kim YD, Oh YL, Seto M, Ko YH (2007). Genome-wide array-based comparative genomic hybridization of ocular marginal zone B cell lymphoma: comparison with pulmonary and nodal marginal zone B cell lymphoma. Genes, Chromosom Cancer.

[81] Honma K, Tsuzuki S, Nakagawa M, Karnan S, Aizawa Y, Kim WS, Kim YD, Ko YH, Seto M (2008). TNFAIP3 is the target gene of chromosome band 6q23.3-q24.1 loss in ocular adnexal marginal zone B cell lymphoma. Genes Chromosom Cancer.

[82] Chanudet E, Ye H, Ferry J, Bacon CM, Adam P, Muller-Hermelink HK, Radford J, Pileri SA, Ichimura K, Collins VP, Hamoudi RA, Nicholson AG, Wotherspoon AC, Isaacson PG, Du MQ (2009). A20 deletion is associated with copy number gain at the TNFA/B/C locus and occurs preferentially in translocation-negative MALT lymphoma of the ocular adnexa and salivary glands. J Pathol.

[83] Kwee I, Rancoita PM, Rinaldi A, Ferreri AJ, Bhagat G, Gascoyne RD, Canzonieri V, Gaidano G, Doglioni C, Zucca E, Ponzoni M, Bertoni F (2011). Genomic profiles of MALT lymphomas: variability across anatomical sites. Haematologica.

[84] Go JH, Yang WI, Ree HJ (2001). Mutational analysis of the 5′ noncoding region of the bcl-6 gene in primary gastric lymphomas. Mod Pathol.

[85] Deutsch AJ, Fruhwirth M, Aigelsreiter A, Cerroni L, Neumeister P (2009). Primary cutaneous marginal zone B-cell lymphomas are targeted by aberrant somatic hypermutation. J Investig Dermatol.

[86] Deutsch AJ, Aigelsreiter A, Staber PB, Beham A, Linkesch W, Guelly C, Brezinschek RI, Fruhwirth M, Emberger W, Buettner M, Beham-Schmid C, Neumeister P (2007). MALT lymphoma and extranodal diffuse large B-cell lymphoma are targeted by aberrant somatic hypermutation. Blood.

[87] Yan Q, Wang M, Moody S, Xue X, Huang Y, Bi Y, Du MQ (2013). Distinct involvement of NF-kappaB regulators by somatic mutation in ocular adnexal malt lymphoma. Br J Haematol.

[88] Li ZM, Rinaldi A, Cavalli A, Mensah AA, Ponzoni M, Gascoyne RD, Bhagat G, Zucca E, Bertoni F (2012). MYD88 somatic mutations in MALT lymphomas. Br J Haematol.

[89] Johansson P, Klein-Hitpass L, Grabellus F, Arnold G, Klapper W, Pfortner R, Duhrsen U, Eckstein A, Durig J, Kuppers R (2016) Recurrent mutations in NF-kappaB pathway components, KMT2D, and NOTCH1/2 in ocular adnexal MALT-type marginal zone lymphomas. Oncotarget. 10.18632/oncotarget.11548 10.18632/oncotarget.11548 PMC5308752 27566587

[90] Du MQ, Peng H, Liu H, Hamoudi RA, Diss TC, Willis TG, Ye H, Dogan A, Wotherspoon AC, Dyer MJ, Isaacson PG (2000). BCL10 gene mutation in lymphoma. Blood.

[91] Jung H, Yoo HY, Lee SH, Shin S, Kim SC, Lee S, Joung JG, Nam JY, Ryu D, Yun JW, Choi JK, Ghosh A, Kim KK, Kim SJ, Kim WS, Park WY, Ko YH (2017). The mutational landscape of ocular marginal zone lymphoma identifies frequent alterations in TNFAIP3 followed by mutations in TBL1XR1 and CREBBP. Oncotarget.

[92] Oeckinghaus A, Ghosh S (2009). The NF-kappaB family of transcription factors and its regulation. Cold Spring Harb Perspect Biol.

[93] Motshwene PG, Moncrieffe MC, Grossmann JG, Kao C, Ayaluru M, Sandercock AM, Robinson CV, Latz E, Gay NJ (2009). An oligomeric signaling platform formed by the Toll-like receptor signal transducers MyD88 and IRAK-4. J Biol Chem.

[94] Rosebeck S, Rehman AO, Lucas PC, McAllister-Lucas LM (2011). From MALT lymphoma to the CBM signalosome: three decades of discovery. Cell Cycle.

[95] Kanayama A, Seth RB, Sun L, Ea CK, Hong M, Shaito A, Chiu YH, Deng L, Chen ZJ (2004). TAB2 and TAB3 activate the NF-kappaB pathway through binding to polyubiquitin chains. Mol Cell.

[96] Jaworski M, Thome M (2016). The paracaspase MALT1: biological function and potential for therapeutic inhibition. Cell Mol Life Sci: CMLS.

[97] Afonina IS, Elton L, Carpentier I, Beyaert R (2015). MALT1—a universal soldier: multiple strategies to ensure NF-kappaB activation and target gene expression. FEBS J.

[98] Chanudet E, Huang Y, Ichimura K, Dong G, Hamoudi RA, Radford J, Wotherspoon AC, Isaacson PG, Ferry J, Du MQ (2010). A20 is targeted by promoter methylation, deletion and inactivating mutation in MALT lymphoma. Leukemia.

[99] Elton L, Carpentier I, Staal J, Driege Y, Haegman M, Beyaert R (2016). MALT1 cleaves the E3 ubiquitin ligase HOIL-1 in activated T cells, generating a dominant negative inhibitor of LUBAC-induced NF-kappaB signaling. FEBS J.

[100] Klein T, Fung SY, Renner F, Blank MA, Dufour A, Kang S, Bolger-Munro M, Scurll JM, Priatel JJ, Schweigler P, Melkko S, Gold MR, Viner RI, Regnier CH, Turvey SE, Overall CM (2015). The paracaspase MALT1 cleaves HOIL1 reducing linear ubiquitination by LUBAC to dampen lymphocyte NF-kappaB signalling. Nat Commun.

[101] Rosebeck S, Lim MS, Elenitoba-Johnson KS, McAllister-Lucas LM, Lucas PC (2016). API2-MALT1 oncoprotein promotes lymphomagenesis via unique program of substrate ubiquitination and proteolysis. World J Biol Chem.

[102] Stoffel A, Le Beau MM (2001). The API2/MALT1 fusion product may lead to germinal center B cell lymphomas by suppression of apoptosis. Human Hered.

[103] Dierlamm J, Baens M, Wlodarska I, Stefanova-Ouzounova M, Hernandez JM, Hossfeld DK, De Wolf-Peeters C, Hagemeijer A, Van den Berghe H, Marynen P (1999). The apoptosis inhibitor gene API2 and a novel 18q gene, MLT, are recurrently rearranged in the t(11;18)(q21;q21) associated with mucosa-associated lymphoid tissue lymphomas. Blood.

[104] Rosebeck S, Madden L, Jin X, Gu S, Apel IJ, Appert A, Hamoudi RA, Noels H, Sagaert X, Van Loo P, Baens M, Du MQ, Lucas PC, McAllister-Lucas LM (2011). Cleavage of NIK by the API2-MALT1 fusion oncoprotein leads to noncanonical NF-kappaB activation. Science.

[105] Nie Z, Du MQ, McAllister-Lucas LM, Lucas PC, Bailey NG, Hogaboam CM, Lim MS, Elenitoba-Johnson KS (2015). Conversion of the LIMA1 tumour suppressor into an oncogenic LMO-like protein by API2-MALT1 in MALT lymphoma. Nat Commun.

[106] Conconi A, Franceschetti S, Aprile von Hohenstaufen K, Margiotta-Casaluci G, Stathis A, Moccia AA, Bertoni F, Ramponi A, Mazzucchelli L, Cavalli F, Gaidano G, Zucca E (2015). Histologic transformation in marginal zone lymphomasdagger. Ann Oncol.

[107] Zucca E, Conconi A, Pedrinis E, Cortelazzo S, Motta T, Gospodarowicz MK, Patterson BJ, Ferreri AJ, Ponzoni M, Devizzi L, Giardini R, Pinotti G, Capella C, Zinzani PL, Pileri S, Lopez-Guillermo A, Campo E, Ambrosetti A, Baldini L, Cavalli F, International Extranodal Lymphoma Study G (2003). Nongastric marginal zone B-cell lymphoma of mucosa-associated lymphoid tissue. Blood.

[108] Maeshima AM, Taniguchi H, Toyoda K, Yamauchi N, Makita S, Fukuhara S, Munakata W, Maruyama D, Kobayashi Y, Tobinai K (2016). Clinicopathological features of histological transformation from extranodal marginal zone B-cell lymphoma of mucosa-associated lymphoid tissue to diffuse large B-cell lymphoma: an analysis of 467 patients. Br J Haematol.

[109] Schreuder MI, Hoeve MA, Hebeda KM, Verdijk MA, Ligtenberg MJ, Bot FJ, Chott A, van Krieken JH (2003). Mutual exclusion of t(11;18)(q21;q21) and numerical chromosomal aberrations in the development of different types of primary gastric lymphomas. Br J Haematol.

[110] Starostik P, Patzner J, Greiner A, Schwarz S, Kalla J, Ott G, Muller-Hermelink HK (2002). Gastric marginal zone B-cell lymphomas of MALT type develop along 2 distinct pathogenetic pathways. Blood.

[111] Toracchio S, Ota H, de Jong D, Wotherspoon A, Rugge M, Graham DY, Samani A, El-Zimaity HM (2009). Translocation t(11;18)(q21;q21) in gastric B-cell lymphomas. Cancer Sci.

[112] Huang X, Zhang Z, Liu H, Ye H, Chuang SS, Wang J, Lin S, Gao Z, Du MQ (2003). t(11;18)(q21;q21) in gastric MALT lymphoma and diffuse large B-cell lymphoma of Chinese patients. Hematol J.

[113] Du M, Peng H, Singh N, Isaacson PG, Pan L (1995). The accumulation of p53 abnormalities is associated with progression of mucosa-associated lymphoid tissue lymphoma. Blood.

[114] Neumeister P, Hoefler G, Beham-Schmid C, Schmidt H, Apfelbeck U, Schaider H, Linkesch W, Sill H (1997). Deletion analysis of the p16 tumor suppressor gene in gastrointestinal mucosa-associated lymphoid tissue lymphomas. Gastroenterology.

[115] Chen YW, Liang AC, Au WY, Chu KM, Wong KY, Hu X, Lu L, Tang JC, Chan KW, Beh SL, Kwong YL, Liang RH, Srivastava G (2003). Multiple BCL6 translocation partners in individual cases of gastric lymphoma. Blood.

[116] Liang R, Chan WP, Kwong YL, Xu WS, Srivastava G, Ho FC (1997). High incidence of BCL-6 gene rearrangement in diffuse large B-cell lymphoma of primary gastric origin. Cancer Genet Cytogenet.

[117] Flossbach L, Antoneag E, Buck M, Siebert R, Mattfeldt T, Moller P, Barth TF (2011). BCL6 gene rearrangement and protein expression are associated with large cell presentation of extranodal marginal zone B-cell lymphoma of mucosa-associated lymphoid tissue. Int J Cancer.

[118] Sonoki T, Harder L, Horsman DE, Karran L, Taniguchi I, Willis TG, Gesk S, Steinemann D, Zucca E, Schlegelberger B, Sole F, Mungall AJ, Gascoyne RD, Siebert R, Dyer MJ (2001). Cyclin D3 is a target gene of t(6;14)(p21.1;q32.3) of mature B-cell malignancies. Blood.

[119] Huang W, Guo L, Liu H, Zheng B, Ying J, Lv N (2014). C-MYC overexpression predicts aggressive transformation and a poor outcome in mucosa-associated lymphoid tissue lymphomas. Int J Clin Exp Pathol.

[120] Sagaert X, de Paepe P, Libbrecht L, Vanhentenrijk V, Verhoef G, Thomas J, Wlodarska I, De Wolf-Peeters C (2006). Forkhead box protein P1 expression in mucosa-associated lymphoid tissue lymphomas predicts poor prognosis and transformation to diffuse large B-cell lymphoma. J Clin Oncol Off J Am Soc Clin Oncol.

[121] Deutsch AJ, Steinbauer E, Hofmann NA, Strunk D, Gerlza T, Beham-Schmid C, Schaider H, Neumeister P (2013). Chemokine receptors in gastric MALT lymphoma: loss of CXCR4 and upregulation of CXCR7 is associated with progression to diffuse large B-cell lymphoma. Mod Pathol.

[122] Kanellis G, Roncador G, Arribas A, Mollejo M, Montes-Moreno S, Maestre L, Campos-Martin Y, Rios Gonzalez JL, Martinez-Torrecuadrada JL, Sanchez-Verde L, Pajares R, Cigudosa JC, Martin MC, Piris MA (2009). Identification of MNDA as a new marker for nodal marginal zone lymphoma. Leukemia.

[123] Metcalf RA, Monabati A, Vyas M, Roncador G, Gualco G, Bacchi CE, Younes SF, Natkunam Y, Freud AG (2014). Myeloid cell nuclear differentiation antigen is expressed in a subset of marginal zone lymphomas and is useful in the differential diagnosis with follicular lymphoma. Hum Pathol.

[124] Falini B, Agostinelli C, Bigerna B, Pucciarini A, Pacini R, Tabarrini A, Falcinelli F, Piccioli M, Paulli M, Gambacorta M, Ponzoni M, Tiacci E, Ascani S, Martelli MP, Dalla Favera R, Stein H, Pileri SA (2012). IRTA1 is selectively expressed in nodal and extranodal marginal zone lymphomas. Histopathology.

[125] Ikeda JI, Kohara M, Tsuruta Y, Nojima S, Tahara S, Ohshima K, Kurashige M, Wada N, Morii E (2017). Immunohistochemical analysis of the novel marginal zone B-cell marker IRTA1 in malignant lymphoma. Hum Pathol.

[126] Ye H, Gong L, Liu H, Hamoudi RA, Shirali S, Ho L, Chott A, Streubel B, Siebert R, Gesk S, Martin-Subero JI, Radford JA, Banerjee S, Nicholson AG, Ranaldi R, Remstein ED, Gao Z, Zheng J, Isaacson PG, Dogan A, Du MQ (2005). MALT lymphoma with t(14;18)(q32;q21)/IGH-MALT1 is characterized by strong cytoplasmic MALT1 and BCL10 expression. J Pathol.

[127] Sagaert X, Laurent M, Baens M, Wlodarska I, De Wolf-Peeters C (2006). MALT1 and BCL10 aberrations in MALT lymphomas and their effect on the expression of BCL10 in the tumour cells. Mod Pathol.

[128] van Krieken JH, Langerak AW, Macintyre EA, Kneba M, Hodges E, Sanz RG, Morgan GJ, Parreira A, Molina TJ, Cabecadas J, Gaulard P, Jasani B, Garcia JF, Ott M, Hannsmann ML, Berger F, Hummel M, Davi F, Bruggemann M, Lavender FL, Schuuring E, Evans PA, White H, Salles G, Groenen PJ, Gameiro P, Pott C, Dongen JJ (2007). Improved reliability of lymphoma diagnostics via PCR-based clonality testing: report of the BIOMED-2 Concerted Action BHM4-CT98-3936. Leukemia.

[129] Evans PA, Pott C, Groenen PJ, Salles G, Davi F, Berger F, Garcia JF, van Krieken JH, Pals S, Kluin P, Schuuring E, Spaargaren M, Boone E, Gonzalez D, Martinez B, Villuendas R, Gameiro P, Diss TC, Mills K, Morgan GJ, Carter GI, Milner BJ, Pearson D, Hummel M, Jung W, Ott M, Canioni D, Beldjord K, Bastard C, Delfau-Larue MH, van Dongen JJ, Molina TJ, Cabecadas J (2007). Significantly improved PCR-based clonality testing in B-cell malignancies by use of multiple immunoglobulin gene targets. Report of the BIOMED-2 Concerted Action BHM4-CT98-3936. Leukemia.

[130] van Maldegem F, Wormhoudt TA, Mulder MM, Oud ME, Schilder-Tol E, Musler AR, Aten J, Saeed P, Kersten MJ, Pals ST, van Noesel CJ, Bende RJ (2012). Chlamydia psittaci-negative ocular adnexal marginal zone B-cell lymphomas have biased VH4-34 immunoglobulin gene expression and proliferate in a distinct inflammatory environment. Leukemia.

[131] Miklos JA, Swerdlow SH, Bahler DW (2000). Salivary gland mucosa-associated lymphoid tissue lymphoma immunoglobulin V(H) genes show frequent use of V1-69 with distinctive CDR3 features. Blood.

[132] Michaeli M, Tabibian-Keissar H, Schiby G, Shahaf G, Pickman Y, Hazanov L, Rosenblatt K, Dunn-Walters DK, Barshack I, Mehr R (2014). Immunoglobulin gene repertoire diversification and selection in the stomach—from gastritis to gastric lymphomas. Front Immunol.

[133] Schreuder MI, Hoefnagel JJ, Jansen PM, van Krieken JH, Willemze R, Hebeda KM (2005). FISH analysis of MALT lymphoma-specific translocations and aneuploidy in primary cutaneous marginal zone lymphoma. J Pathol.

[134] Ventura RA, Martin-Subero JI, Jones M, McParland J, Gesk S, Mason DY, Siebert R (2006). FISH analysis for the detection of lymphoma-associated chromosomal abnormalities in routine paraffin-embedded tissue. J Mol Diagn: JMD.

[135] Dierlamm J, Baens M, Stefanova-Ouzounova M, Hinz K, Wlodarska I, Maes B, Steyls A, Driessen A, Verhoef G, Gaulard P, Hagemeijer A, Hossfeld DK, De Wolf-Peeters C, Marynen P (2000). Detection of t(11;18)(q21;q21) by interphase fluorescence in situ hybridization using API2 and MLT specific probes. Blood.

[136] Remstein ED, Kurtin PJ, James CD, Wang XY, Meyer RG, Dewald GW (2002). Mucosa-associated lymphoid tissue lymphomas with t(11;18)(q21;q21) and mucosa-associated lymphoid tissue lymphomas with aneuploidy develop along different pathogenetic pathways. Am J Pathol.

[137] Inagaki H, Okabe M, Seto M, Nakamura S, Ueda R, Eimoto T (2001). API2-MALT1 fusion transcripts involved in mucosa-associated lymphoid tissue lymphoma: multiplex RT-PCR detection using formalin-fixed paraffin-embedded specimens. Am J Pathol.

[138] Schreuder MI, Hoeve MA, Groothuis L, Boot H, Boerrigter LH, de Jong D, Veenendaal RA, Jansen JH, van Krieken JH (2005). Monitoring gastric lymphoma in peripheral blood by quantitative IgH allele-specific oligonucleotide real-time PCR and API2-MALT1 PCR. Br J Haematol.

[139] Ferreri AJ, Govi S, Pasini E, Mappa S, Bertoni F, Zaja F, Montalban C, Stelitano C, Cabrera ME, Giordano Resti A, Politi LS, Doglioni C, Cavalli F, Zucca E, Ponzoni M, Dolcetti R (2012). Chlamydophila psittaci eradication with doxycycline as first-line targeted therapy for ocular adnexae lymphoma: final results of an international phase II trial. J Clin Oncol Off J Am Soc Clin Oncol.

[140] Wundisch T, Thiede C, Morgner A, Dempfle A, Gunther A, Liu H, Ye H, Du MQ, Kim TD, Bayerdorffer E, Stolte M, Neubauer A (2005). Long-term follow-up of gastric MALT lymphoma after Helicobacter pylori eradication. J Clin Oncol: Off J Am Soci Clin Oncol.

[141] Zullo A, Hassan C, Cristofari F, Andriani A, De Francesco V, Ierardi E, Tomao S, Stolte M, Morini S, Vaira D (2010). Effects of Helicobacter pylori eradication on early stage gastric mucosa-associated lymphoid tissue lymphoma. Clin Gastroenterol Hepatol.

[142] Nakamura S, Sugiyama T, Matsumoto T, Iijima K, Ono S, Tajika M, Tari A, Kitadai Y, Matsumoto H, Nagaya T, Kamoshida T, Watanabe N, Chiba T, Origasa H, Asaka M, Group JGS (2012). Long-term clinical outcome of gastric MALT lymphoma after eradication of Helicobacter pylori: a multicentre cohort follow-up study of 420 patients in Japan. Gut.

[143] Gong EJ, Ahn JY, Jung HY, Park H, Ko YB, Na HK, Jung KW, Kim DH, Lee JH, Choi KD, Song HJ, Lee GH, Kim JH (2016) Helicobacter pylori eradication therapy is effective as the initial treatment for patients with H. pylori -negative and disseminated gastric mucosa-associated lymphoid tissue lymphoma. Gut Liver. 10.5009/gnl15510 10.5009/gnl15510 PMC5003192 27114423

[144] Raderer M, Jager G, Brugger S, Puspok A, Fiebiger W, Drach J, Wotherspoon A, Chott A (2003). Rituximab for treatment of advanced extranodal marginal zone B cell lymphoma of the mucosa-associated lymphoid tissue lymphoma. Oncology.

[145] Kiesewetter B, Troch M, Dolak W, Mullauer L, Lukas J, Zielinski CC, Raderer M (2013). A phase II study of lenalidomide in patients with extranodal marginal zone B-cell lymphoma of the mucosa associated lymphoid tissue (MALT lymphoma). Haematologica.

[146] ZuccaE, ConconiA, MartinelliG, BouabdallahR, TucciA, VitoloU, MartelliM, PettengellR, SallesG, SebbanC, GuillermoAL, PinottiG, DevizziL, MorschhauserF, TillyH, TorriV, HohausS, FerreriAJ, ZacheeP, BoslyA, HaiounC, StelitanoC, BelleiM, PonzoniM, Copie-BergmanC, JackA, CampoE, MazzucchelliL, CavalliF, JohnsonP, ThieblemontC (2017) Final results of the IELSG-19 randomized trial of mucosa-associated lymphoid tissue lymphoma: improved event-free and progression-free survival with rituximab plus chlorambucil versus either chlorambucil or rituximab monotherapy. Journal of Clinical Oncology: official journal of the American Society of Clinical Oncology:JCO2016706994. doi:10.1200/JCO.2016.70.6994 10.1200/JCO.2016.70.6994 28355112

[147] Kiesewetter B, Willenbacher E, Willenbacher W, Egle A, Neumeister P, Voskova D, Mayerhoefer ME, Simonitsch-Klupp I, Melchardt T, Greil R, Raderer M (2016) A phase II study of rituximab plus lenalidomide for mucosa-associated lymphoid tissue lymphoma (MALT lymphoma). Blood. 10.1182/blood-2016-06-720599 10.1182/blood-2016-06-720599 27879257

[148] Rosenthal A, Dueck AC, Ansell S, Gano K, Conley C, Nowakowski GS, Camoriano J, Leis JF, Mikhael JR, Keith Stewart A, Inwards D, Dingli D, Kumar S, Noel P, Gertz M, Porrata L, Russell S, Colgan J, Fonseca R, Habermann TM, Kapoor P, Buadi F, Leung N, Tiedemann R, Witzig TE, Reeder C (2017). A phase 2 study of lenalidomide, rituximab, cyclophosphamide, and dexamethasone (LR-CD) for untreated low-grade non-Hodgkin lymphoma requiring therapy. Am J Hematol.

[149] Conconi A, Raderer M, Franceschetti S, Devizzi L, Ferreri AJ, Magagnoli M, Arcaini L, Zinzani PL, Martinelli G, Vitolo U, Kiesewetter B, Porro E, Stathis A, Gaidano G, Cavalli F, Zucca E (2014). Clinical activity of everolimus in relapsed/refractory marginal zone B-cell lymphomas: results of a phase II study of the International Extranodal Lymphoma Study Group. Br J Haematol.

[150] Chen R, Frankel P, Popplewell L, Siddiqi T, Ruel N, Rotter A, Thomas SH, Mott M, Nathwani N, Htut M, Nademanee A, Forman SJ, Kirschbaum M (2015). A phase II study of vorinostat and rituximab for treatment of newly diagnosed and relapsed/refractory indolent non-Hodgkin lymphoma. Haematologica.

[151] Kirschbaum MH, Goldman BH, Zain JM, Cook JR, Rimsza LM, Forman SJ, Fisher RI (2012). A phase 2 study of vorinostat for treatment of relapsed or refractory Hodgkin lymphoma: Southwest Oncology Group Study S0517. Leuk Lymphoma.

[152] Conconi A, Martinelli G, Lopez-Guillermo A, Zinzani PL, Ferreri AJ, Rigacci L, Devizzi L, Vitolo U, Luminari S, Cavalli F, Zucca E, International Extranodal Lymphoma Study G (2011). Clinical activity of bortezomib in relapsed/refractory MALT lymphomas: results of a phase II study of the International Extranodal Lymphoma Study Group (IELSG). Ann Oncol.

[153] Advani RH, Buggy JJ, Sharman JP, Smith SM, Boyd TE, Grant B, Kolibaba KS, Furman RR, Rodriguez S, Chang BY, Sukbuntherng J, Izumi R, Hamdy A, Hedrick E, Fowler NH (2013). Bruton tyrosine kinase inhibitor ibrutinib (PCI-32765) has significant activity in patients with relapsed/refractory B-cell malignancies. J Clin Oncol: Off J Am Soc Clin Oncol.

[154] Noy A, de Vos S, Thieblemont C, Martin P, Flowers CR, Morschhauser F, Collins GP, Ma S, Coleman M, Peles S, Smith S, Barrientos JC, Smith A, Munneke B, Dimery I, Beaupre DM, Chen R (2017). Targeting Bruton tyrosine kinase with ibrutinib in relapsed/refractory marginal zone lymphoma. Blood.

[155] Gopal AK, Kahl BS, de Vos S, Wagner-Johnston ND, Schuster SJ, Jurczak WJ, Flinn IW, Flowers CR, Martin P, Viardot A, Blum KA, Goy AH, Davies AJ, Zinzani PL, Dreyling M, Johnson D, Miller LL, Holes L, Li D, Dansey RD, Godfrey WR, Salles GA (2014). PI3Kdelta inhibition by idelalisib in patients with relapsed indolent lymphoma. N Engl J Med.

[156] Ferch U, Kloo B, Gewies A, Pfander V, Duwel M, Peschel C, Krappmann D, Ruland J (2009). Inhibition of MALT1 protease activity is selectively toxic for activated B cell-like diffuse large B cell lymphoma cells. J Exp Med.

[157] Fontan L, Yang C, Kabaleeswaran V, Volpon L, Osborne MJ, Beltran E, Garcia M, Cerchietti L, Shaknovich R, Yang SN, Fang F, Gascoyne RD, Martinez-Climent JA, Glickman JF, Borden K, Wu H, Melnick A (2012). MALT1 small molecule inhibitors specifically suppress ABC-DLBCL in vitro and in vivo. Cancer Cell.

[158] Nagel D, Spranger S, Vincendeau M, Grau M, Raffegerst S, Kloo B, Hlahla D, Neuenschwander M, Peter von Kries J, Hadian K, Dorken B, Lenz P, Lenz G, Schendel DJ, Krappmann D (2012). Pharmacologic inhibition of MALT1 protease by phenothiazines as a therapeutic approach for the treatment of aggressive ABC-DLBCL. Cancer Cell.

